# Within-patient evolution of *Pseudomonas aeruginosa* populations during antimicrobial treatment

**DOI:** 10.1128/msphere.00656-25

**Published:** 2026-03-16

**Authors:** Giuseppe Fleres, Ellen G. Kline, Kevin M. Squires, Tyler Tate, Hannah M. Creager, Ryan K. Shields, Daria Van Tyne

**Affiliations:** 1 Division of Infectious Diseases, University of Pittsburgh School of Medicine 12317 , Pittsburgh, Pennsylvania, USA; 2 Center for Evolutionary Biology and Medicine, University of Pittsburgh School of Medicine 12317 , Pittsburgh, Pennsylvania, USA; 3 Center for Innovative Antimicrobial Therapy, University of Pittsburgh School of Medicine 12317 , Pittsburgh, Pennsylvania, USA; 4 Antibiotic Management Program, University of Pittsburgh Medical Center 6595, Pittsburgh, Pennsylvania, USA; Third Institute of Oceanography, Xiamen, China

**Keywords:** culture-enriched metagenomics, *Pseudomonas aeruginosa*, within-host evolution

## Abstract

Multidrug-resistant (MDR) *Pseudomonas aeruginosa* infections pose a major challenge to effective treatment. Understanding genomic adaptations during antimicrobial therapy in patients infected with this pathogen is crucial for preventing therapeutic failure. Here, we investigated the population diversity and evolution of *P. aeruginosa* collected longitudinally from six patients who evolved multidrug-resistant infections. Serial clinical *P. aeruginosa* single-colony isolates (*n* = 63) and culture-enriched metagenomic population samples (*n* = 39) were collected and subjected to whole-genome sequencing. The resulting data were used to characterize and compare the species composition, multi-locus sequence types (STs), and resistance-associated mutations present within each sample type. Single-colony isolate sequencing showed that each patient was infected with a single *P. aeruginosa* strain that accumulated mutations and became increasingly more antibiotic-resistant over time. Mutations in genes associated with β-lactam resistance, including *ampC, ftsI*, and *mexR,* arose over time and corresponded with changes in antimicrobial susceptibility in single-colony isolates. Species profiling of culture-enriched metagenomic populations revealed that all samples contained *P. aeruginosa*, but also additional gram-negative pathogens. Metagenomic analysis of culture-enriched populations identified resistance-associated mutations at low frequency, many of which were not identified in single-colony isolates from the same sample. In some cases, resistance-associated mutations initially detected at low frequency rose to fixation after antimicrobial treatment. Overall, this study shows that population-based metagenomic sequencing effectively captures the within-patient genomic diversity of *P. aeruginosa* during antimicrobial therapy and could aid the detection and interpretation of resistance-associated mutations in this pathogen.

**IMPORTANCE:**

*Pseudomonas aeruginosa* infections are notoriously difficult to treat and are associated with high rates of morbidity and mortality. While the genetic basis of resistance in *P. aeruginosa* is well documented *in vitro*, less is known about how resistance evolves within patients during antibiotic therapy. Standard approaches based on analysis of clonal isolates may miss within-patient diversity, potentially overlooking low-frequency mutations that contribute to treatment failure. In this study, we compared single-colony isolate whole-genome sequencing with culture-enriched metagenomic sequencing to monitor the evolution of *P. aeruginosa* populations in patients receiving antibiotic therapy. The culture-enriched metagenomic approach enabled the detection of emerging resistance mutations, such as low-frequency variants in *ampC* and *ftsI*, before these variants rose to fixation. It also revealed genetically resistant subpopulations missed by isolate sequencing alone. Overall, our findings highlight the value of population-based metagenomic sequencing in capturing bacterial adaptation during infection and underscore its potential to improve resistance surveillance and guide personalized antimicrobial therapy.

## OBSERVATION

*Pseudomonas aeruginosa* is recognized as a serious public health threat by the Centers for Disease Control and Prevention and has been classified by the World Health Organization as a critical priority pathogen, emphasizing the urgent need for new antimicrobial therapies (1). A variety of intrinsic resistance mechanisms, combined with a remarkable capacity to acquire additional resistance determinants, make *P. aeruginosa* infections increasingly difficult to treat (2). Infections caused by multidrug-resistant (MDR) strains are particularly concerning and often necessitate the use of newer β-lactam/β-lactamase inhibitor combinations or siderophore cephalosporins to manage (3, 4). However, the efficacy of these agents is frequently undermined by the emergence of diverse and often convergent resistance mechanisms. These include upregulation of multidrug efflux systems (e.g., MexAB-OprM), inactivation or loss of porins (OprD), mutations affecting the catalytic activity and expression of the chromosomal β-lactamase (AmpC), and point mutations in essential antibiotic target genes like *ftsI*, *gyrA*, and *gyrB* (4–6). Alterations in regulatory genes such as *lasR* further contribute to phenotypic heterogeneity by modulating quorum sensing pathways, which can affect antibiotic tolerance and persistence (2). These adaptive mutations frequently arise under selective therapeutic pressures, facilitating rapid within-host evolution and complicating effective treatment (6).

Although resistance mechanisms in *P. aeruginosa* have been well characterized *in vitro* and among serial clinical isolates collected from infected patients (7, 8), much less is known about how infecting *P. aeruginosa* populations evolve within patients during antibiotic therapy (9). Treatment decisions are typically guided by antimicrobial susceptibility testing and whole-genome sequencing analysis of a single bacterial clone, assuming that it reflects the entire infecting population. However, this approach offers limited insight into within-host diversity and often misses low-frequency variants that may influence treatment outcomes (10, 11). Culture-enriched metagenomic sequencing addresses these limitations by pooling all colonies from a selective culture plate and performing shotgun metagenomic sequencing, enabling a more comprehensive analysis of population-level diversity and evolution during infection (10, 12).

**Fig 1:**
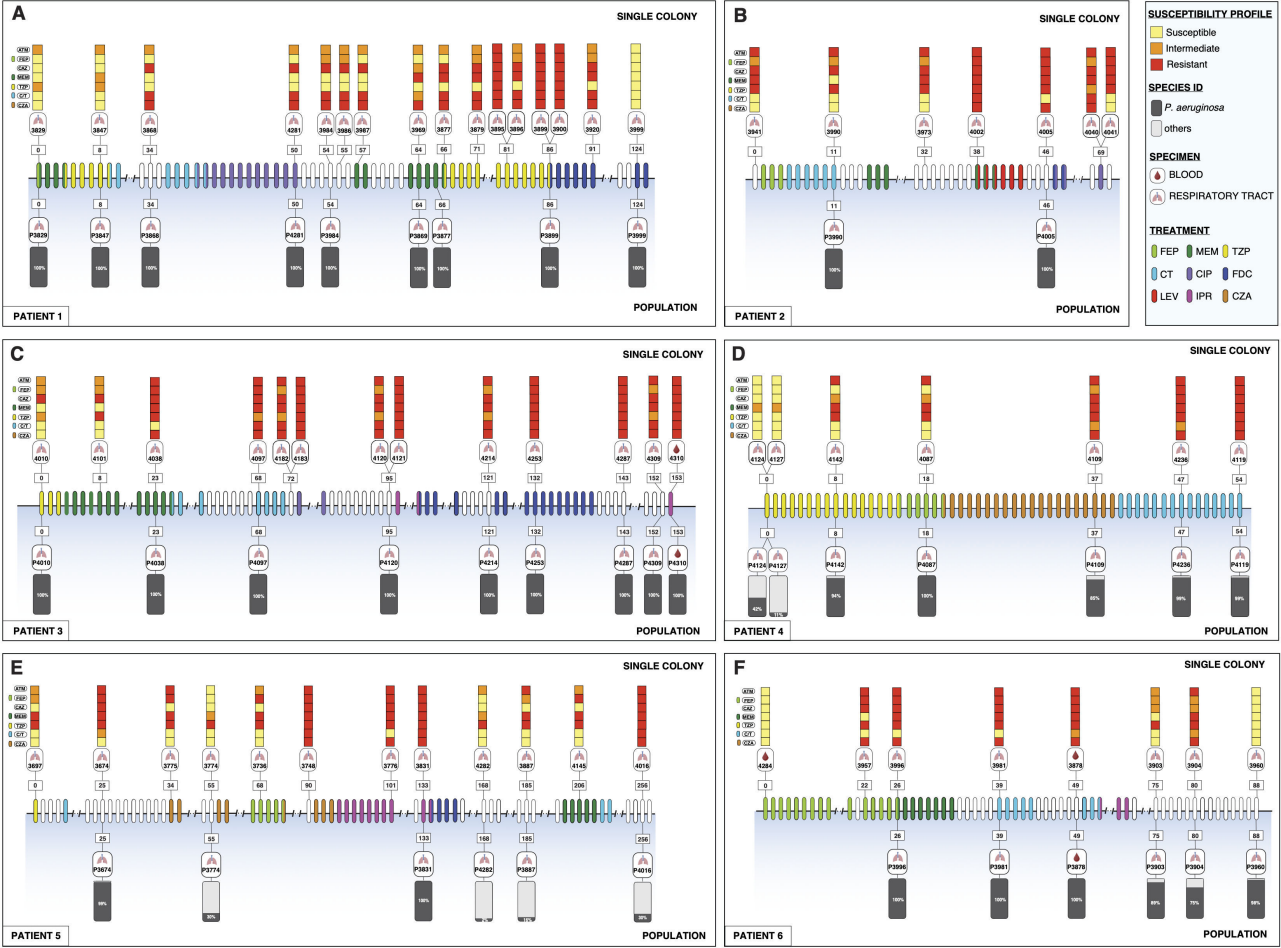
(**A–F**) Timeline of *Pseudomonas aeruginosa* infection and treatment for six patients. Each panel shows longitudinal sampling of *P. aeruginosa* single-colony isolates and culture-enriched metagenomic populations during recurrent infection in a different patient. The upper section of each panel depicts single-colony isolates, while the lower section shows corresponding population samples. Colored squares indicate the antimicrobial susceptibility profile of each single-colony isolate, and gray bars show the proportion of each population composed of *P. aeruginosa* or other bacterial species. β-Lactam antibiotics tested included aztreonam (ATM), cefepime (FEP), ceftazidime (CAZ), meropenem (MEM), piperacillin/tazobactam (TZP), ceftolozane/tazobactam (C/T), and ceftazidime/avibactam (CZA). Ovals in each timeline are colored based on antimicrobial treatment administered to each patient, including FEP, MEM, TZP, C/T, ciprofloxacin (CIP), cefiderocol (FDC), levofloxacin (LEV), imipenem/relebactam (IPR), and CZA. Sample identifiers, source (respiratory tract or blood), and collection time points (in days) are indicated above and below the timeline.

Here, we investigated the within-host evolution of *P. aeruginosa* populations in six patients who developed MDR infections during treatment and compared results obtained from single-colony isolate and culture-enriched metagenomic sequencing. We hypothesized that reliance on single-colony isolate testing would underestimate the genotypic and phenotypic diversity of *P. aeruginosa* within the infected host, particularly under antimicrobial selection. A total of 63 clinical single-colony isolates and 39 culture-enriched metagenomic populations were collected from six patients, sequenced, and analyzed (Fig. 1; File S1; Supplemental text). In all patients, susceptibility profiles of *P. aeruginosa* single-colony isolates progressively shifted toward increasing resistance over the course of treatment. This aligns with prior studies documenting the rapid evolution of resistance in *P. aeruginosa* under antibiotic stress, especially during prolonged therapy in chronic infections (13, 14). In two patients (Patients 1 and 6), single-colony isolates collected at the final time point exhibited a more susceptible phenotype compared to earlier single-colony isolates, suggesting that resistant bacteria may have been outcompeted or replaced, potentially due to changes in treatment, host immunity, or fitness costs associated with resistance (15).

Species profiling of culture-enriched metagenomic samples confirmed the presence of *P. aeruginosa* in all samples (Fig. 1). Metagenomic samples from three patients (Patients 1–3) contained only *P. aeruginosa*, while the other three patients (Patients 4–6) exhibited polymicrobial infections including *P. aeruginosa* and other organisms (Fig. S1). In each case, the same species were identified as part of standard-of-care testing in the clinical microbiology laboratory, suggesting that this approach accurately captured polymicrobial infections.

Single-colony isolate sequencing revealed a *P. aeruginosa* sequence type (ST) that was unique to each patient, and all colonies sequenced from the same patient belonged to the same ST (Fig. S2). Analysis of within-patient single-nucleotide polymorphisms (SNPs) among *P. aeruginosa* single-colony isolates collected from the same patient revealed low genetic diversity in five of the six patients, with pairwise SNP differences ranging from 0 to 23. This narrow range suggests that each patient was infected with a single strain that underwent clonal expansion during infection. In contrast, one patient (Patient 1) displayed markedly higher within-host diversity, with SNP distances reaching up to 411 (Table S2). This elevated level of genetic variation could be attributed to the presence of a hypermutator clone, which accelerates mutation rates and promotes rapid genetic diversification, particularly under selective pressures such as antibiotic treatment and chronic respiratory tract infection (16). Supporting this interpretation, multiple mutations were identified in DNA mismatch repair genes from this patient’s single-colony isolates, including W181R and S324P mutations in *mutS* and an L35P mutation in *mutL*. Although these mutations suggest a possible disruption of mismatch repair function, direct measurement of mutation rates in strains with and without these variants would be required to definitively confirm a hypermutator phenotype.

**Fig 2:**
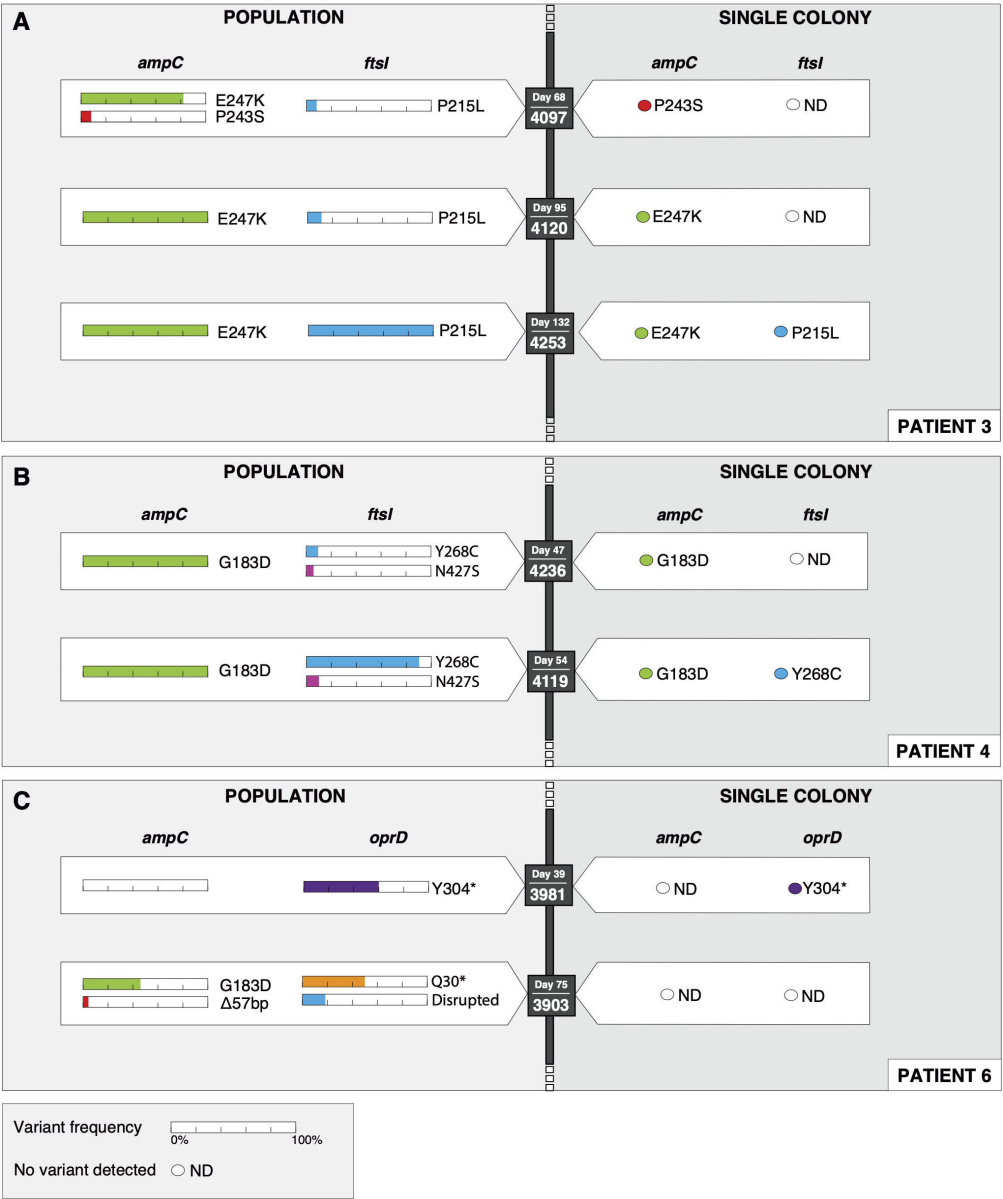
(**A–C**) Comparison of resistance-associated mutations detected in culture-enriched metagenomic populations versus single colonies in three patients with *Pseudomonas aeruginosa* infections. Each panel represents a subset of the single-colony isolate and population data collected from each patient and displays mutations identified in population sequencing (left) and in single-colony isolate whole-genome sequences (right) at corresponding sampling time points. Nonsynonymous mutations, nonsense mutations, and structural variants are shown in β-lactam resistance-associated genes *ampC*, *oprD*, and *ftsI*. Colored bars represent the allele frequency (%) of each mutation in metagenomic populations. Sample ID and collection day are indicated next to each genotype.

We next explored whether culture-enriched metagenomic sequencing could enhance the detection of resistance-associated mutations that might be missed by single-colony isolate sequencing. We performed variant detection using breseq (17), either in consensus mode for single-colony isolates or polymorphism mode for culture-enriched metagenomic populations. Variants with allele frequencies of at least 5% (a cut-off that we reasoned would avoid misclassification of sequencing errors as variants) were included for analysis. Of 11 resistance-associated genes across 6 patients, culture-enriched metagenomic sequencing provided additional insight in 19 out of 66 (29%) cases. In 84% of these cases (*n* = 16/19), this was due to the detection of resistance-associated mutations that were not identified by single-colony isolate sequencing (Table S3). Comparison of single-colony isolate and culture-enriched metagenomic sequencing revealed distinct interpretations depending on the method used (Fig. 2). In Patient 3, single-colony isolate sequencing identified a low-frequency mutation in *ampC* (P243S) but failed to detect a co-occurring mutation (E247K) that was present at over 80% frequency in the population (Fig. 2A). This highlights a key limitation of the single-colony isolate approach, which might randomly capture minority variants while missing the dominant genotype, ultimately leading to an incomplete or misleading resistance profile. In both Patients 3 and 4, mutations in *ftsI* were identified at low frequency in population samples collected at earlier time points, and the same variants were later detected in single-colony isolates as well as in the populations at high frequency (Fig. 2A and B). This illustrates how population sequencing can detect emerging mutations before they become dominant. Finally, in Patient 6, we identified several mutations at allele frequencies less than 50% that were exclusively detected in population samples and were never observed in single-colony isolate genomes (Fig. 2C). Together, these findings emphasize the higher sensitivity of culture-enriched metagenomic sequencing for capturing within-host diversity. They also reveal coexisting subpopulations that might have distinct antibiotic susceptibility phenotypes, representing “hidden diversity” that could influence bacterial persistence and treatment outcomes.

Here, we focused on a targeted set of *P. aeruginosa* genes known to be associated with antibiotic resistance. While this approach allowed for in-depth analysis of clinically relevant resistance mechanisms, it likely underrepresents the broader genomic heterogeneity within infecting *P. aeruginosa* populations. Other adaptive changes (e.g., mutations related to antibiotic tolerance, biofilm formation, and metabolic remodeling) may also contribute to treatment failure but were not systematically assessed here. Conversely, not all mutations in the genes we focused on cause phenotypic resistance or contribute to clinical failure, making it important to cross-reference with known resistance-associated mutations and avoid overinterpretation of unvalidated variants. Furthermore, with only six patients sampled, we were unable to rigorously evaluate how patient-specific factors such as infection site or duration, antimicrobial exposure, host immune status, or the presence of polymicrobial infection might influence evolutionary dynamics. In addition, applying this approach in real time, rather than retrospectively, would be more useful as it might enable early detection of emerging resistance mutations and timely optimization of antimicrobial therapy. The current turnaround time for sample collection, DNA extraction, sequencing, and analysis may not be compatible with acute clinical decision-making, but as whole genome sequencing becomes increasingly utilized in clinical settings, this might be feasible in the future. A recent study using a hybridization-based enrichment panel showed enhanced sensitivity and detection of resistance variants directly from low-biomass clinical specimens, suggesting that targeted metagenomics-based approaches could be adapted to clinical settings (18).

In summary, this study demonstrates that culture-enriched metagenomic sequencing provides enhanced resolution of within-host evolution in *P. aeruginosa* during antimicrobial treatment. We found that population-level sequencing consistently identified low-frequency variants that were missed by single-colony isolate sequencing, revealing a more comprehensive picture of the adaptive landscape in these patients. As microbial next-generation sequencing becomes more commonly used in clinical practice, culture-enriched metagenomic sequencing could provide greater sensitivity for studies of antibiotic resistance and evolutionary dynamics, which could contribute to improved management of patients with difficult-to-treat infections.

## Data Availability

Genome sequencing data used in this study are available in the National Center for Biotechnology Information (NCBI) under BioProject PRJNA1332666, with accession information listed in Table S3.

## SUPPLEMENTAL MATERIAL

File S1 msphere.00656-25-s0001.docx Case summaries.

Supplemental Figures msphere.00656-25-s0002.pdf Figures S1 and S2.

Supplemental text msphere.00656-25-s0003.docx Supplemental methods.

Table S1 msphere.00656-25-s0004.xlsx *P. aeruginosa* genome-wide and individual gene coverage in culture-enriched metagenomic samples.

Table S2 msphere.00656-25-s0005.xlsx Pairwise SNP distance matrices of same-patient *P. aeruginosa* isolates.

Table S3 msphere.00656-25-s0006.xlsx Resistance-associated mutations detected in single-colony and culture-enriched metagenomic data.

ASM does not own the copyrights to Supplemental Material that may be linked to, or accessed through, an article. The authors have granted ASM a non-exclusive, world-wide license to publish the Supplemental Material files. Please contact the corresponding author directly for reuse.
