# Supplementary material for: Within-patient evolution of Pseudomonas aeruginosa populations during antimicrobial treatment
Source: mSphere. 2026 Mar 16;11(4):e00656-25. doi: 10.1128/msphere.00656-25 (PMC13123702; doi:10.1128/msphere.00656-25)
Supplement: Supplemental Figures — Figures S1 and S2. [file msphere.00656-25-s0002.pdf]

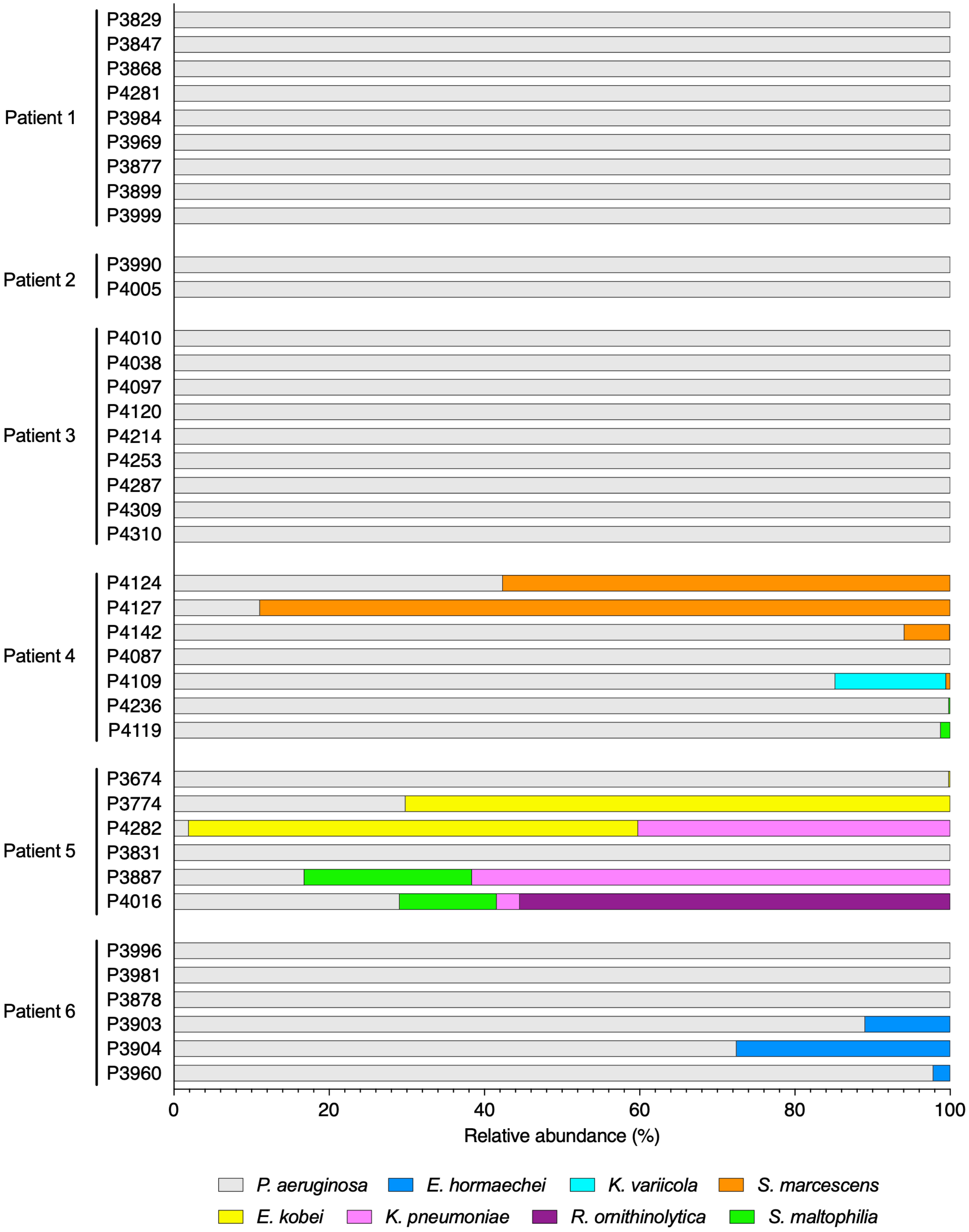

Supplemental Figure 1. Relative abundance of bacterial species identified in culture-enriched metagenomic populations sampled from six patients with *P. aeruginosa* infection. Sequencing reads were assigned to species with Kraken2, and the relative abundance of each species in each sample is shown. Samples are ordered chronologically for each patient, and bars are colored by species.

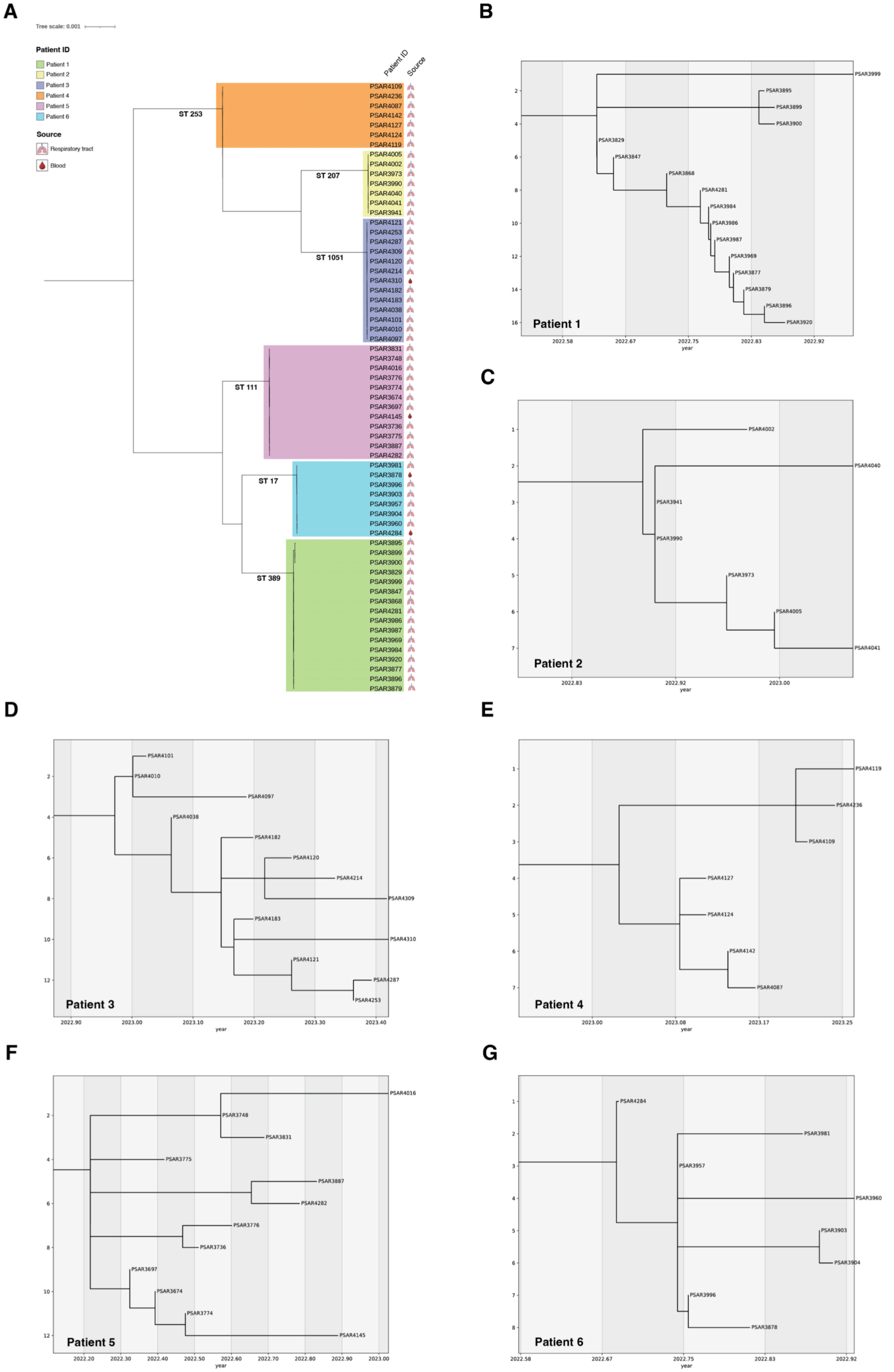

Supplemental Figure 2. (A) Phylogenetic tree of 63 *P. aeruginosa* clinical isolates collected from six patients with *P. aeruginosa* infections. The tree was constructed with RAxML from a reference-free alignment made with SKA. Sequence type (ST) is noted for each branch, and isolates are shaded by patients. (B-G) Time-dated phylogenies made for each patient using the isolates from that patient. Phylogenies were constructed using TimeTree.
