# Supplementary material for: Within-patient evolution of Pseudomonas aeruginosa populations during antimicrobial treatment
Source: mSphere. 2026 Mar 16;11(4):e00656-25. doi: 10.1128/msphere.00656-25 (PMC13123702; doi:10.1128/msphere.00656-25)
Supplement: Supplemental text — Supplemental methods. [file msphere.00656-25-s0003.docx]

**Supplemental Materials and Methods**

**Sample Collection**

Longitudinal clinical specimens were collected from six patients infected with MDR *P. aeruginosa* who developed treatment-associated resistance to beta-lactam agents. For each patient, respiratory and blood culture specimens were plated onto MacConkey agar, and unique *P. aeruginosa* morphologies were identified and reported as part of standard care in the clinical microbiology laboratory. Primary isolation plates were also scraped to pool all colonies on the plate, and were stored as a culture-enriched metagenomic population. This study was approved by the Institutional Review Board at the University of Pittsburgh under STUDY22070065.

**Antimicrobial Susceptibility Testing**

Antimicrobial susceptibility testing was performed using the MicroScan WalkAway system (Beckman Coulter, Brea, CA, USA), and results were interpreted according to Clinical and Laboratory Standards Institute (CLSI) guidelines^1^. To confirm antibiotic resistance for individual isolates, minimum inhibitory concentrations (MICs) of ceftazidime-avibactam (CZA) and ceftolozane-tazobactam (C/T) were determined by broth microdilution in triplicate following CLSI methods.

**Whole-Genome and Culture-Enriched Metagenomic Sequencing and Analysis**

Isolates were subjected to whole-genome sequencing (WGS) on the Illumina platform. Briefly, genomic DNA was extracted using a Qiagen DNeasy Blood and Tissue Kit and short-read libraries were prepared using the Illumina DNA Prep protocol and were sequenced (2 × 150 bp, paired-end reads) on a NextSeq 550. The earliest available single-colony isolate from each patient was used as a patient-specific reference. Assemblies were generated using SPAdes^2^ v3.15.5 and were assessed for quality using QUAST^3^. Species identification and contamination screening were performed with Kraken2^4^ v2.1.3. Multi-locus sequence typing (MLST) was carried out using the PubMLST database via *mlst*^5^ v2.11. Genome annotation was conducted with Prokka^6^ v1.14.5. Pairwise SNP distances and generation of reference-free alignments were computed using Split Kmer Analysis (SKA) v1.0^7^. Resistance-associated mutations were identified from both single-colony isolate and population-derived WGS data using *breseq*^8^. This study focused on a targeted set of 9 genes (Supplemental Table 1) known to be associated with antibiotic resistance in *P. aeruginosa*. For culture-enriched metagenomic samples, *P. aeruginosa* genome-wide and individual gene coverage were calculated, and only samples with ≥100× genome-wide coverage were included in downstream analyses (Supplemental Table 1). A 5% allele frequency cut-off was used to ensure sufficient read support for each variant and to avoid misclassification of sequencing errors as mutations. Phylogenetic trees were constructed using RAxML HPC^9^ v8.2.12 with 100 bootstraps. Time-resolved phylogenies and ancestral sequence reconstruction were performed using TimeTree^10^ v0.11.4.

**References**

1. Clinical and Laboratory Standards Institute (CLSI). Performance Standards for Antimicrobial Susceptibility Testing. in (CLSI, Wayne, PA, 2024).

2. Bankevich, A. *et al.* SPAdes: a new genome assembly algorithm and its applications to single-cell sequencing. *J. Comput. Biol. J. Comput. Mol. Cell Biol.* **19**, 455–477 (2012).

3. Gurevich, A., Saveliev, V., Vyahhi, N. & Tesler, G. QUAST: quality assessment tool for genome assemblies. *Bioinformatics* **29**, 1072–1075 (2013).

4. Wood, D. E., Lu, J. & Langmead, B. Improved metagenomic analysis with Kraken 2. *Genome Biol.* **20**, 257 (2019).

5. Jolley, K. A. & Maiden, M. C. BIGSdb: Scalable analysis of bacterial genome variation at the population level. *BMC Bioinformatics* **11**, 595 (2010).

6. Seemann, T. Prokka: rapid prokaryotic genome annotation. *Bioinformatics* **30**, 2068–2069 (2014).

7. Harris, S. R. SKA: Split Kmer Analysis Toolkit for Bacterial Genomic Epidemiology. 453142 Preprint at https://doi.org/10.1101/453142 (2018).

8. Deatherage, D. E. & Barrick, J. E. Identification of mutations in laboratory-evolved microbes from next-generation sequencing data using breseq. *Methods Mol. Biol. Clifton NJ* **1151**, 165–188 (2014).

9. Stamatakis, A. RAxML version 8: a tool for phylogenetic analysis and post-analysis of large phylogenies. *Bioinformatics* **30**, 1312–1313 (2014).

10. Kumar, S., Stecher, G., Suleski, M. & Hedges, S. B. TimeTree: A Resource for Timelines, Timetrees, and Divergence Times. *Mol. Biol. Evol.* **34**, 1812–1819 (2017).
